# SARS-CoV-2 Neutralizing Antibodies: A Network Meta-Analysis across Vaccines

**DOI:** 10.3390/vaccines9030227

**Published:** 2021-03-05

**Authors:** Paola Rogliani, Alfredo Chetta, Mario Cazzola, Luigino Calzetta

**Affiliations:** 1 Unit of Respiratory Medicine, Department of Experimental Medicine, University of Rome “Tor Vergata”, 00133 Rome, Italy; paola.rogliani@uniroma2.it (P.R.); mario.cazzola@uniroma2.it (M.C.); 2 Respiratory Disease and Lung Function Unit, Department of Medicine and Surgery, University of Parma, 43126 Parma, Italy; alfredoantonio.chetta@unipr.it

**Keywords:** COVID-19, network meta-analysis, neutralizing antibodies, SARS-CoV-2, vaccine

## Abstract

*Background*: There are no studies providing head-to-head comparison across SARS-CoV-2 vaccines. Therefore, we compared the efficacy of candidate vaccines in inducing neutralizing antibodies against SARS-CoV-2. *Methods*: A network meta-analysis was performed to compare the peak levels of SARS-CoV-2 neutralizing antibodies across candidate vaccines. Data were reported as standardized mean difference (SMD) since the outcome was assessed via different metrics and methods across the studies. *Results*: Data obtained from 836 healthy adult vaccine recipients were extracted from 11 studies. BBIBP-CorV, AZD1222, BNT162b2, New Crown COVID-19, and Sputnik V induced a very large effect on the level of neutralizing antibodies (SMD > 1.3); CoVLP, CoronaVac, NVX-CoV2373, and Ad5-nCoV induced a large effect (SMD > 0.8 to ≤1.3); and Ad26.COV2.S induced a medium effect (SMD > 0.5 to ≤0.8). BBIBP-CorV and AZD122 were more effective (*p* < 0.05) than Ad26.COV2.S, Ad5–nCoV, mRNA-1237, CoronaVac, NVX–CoV2373, CoVLP, and New Crown COVID-19; New Crown COVID-19 was more effective (*p* < 0.05) than Ad26.COV2.S, Ad5–nCoV, and mRNA-1237; CoronaVac was more effective (*p* < 0.05) than Ad26.COV2.S and Ad5–nCoV; and Sputnik V and BNT162b2 were more effective (*p* < 0.05) than Ad26.COV2.S. In recipients aged ≤60 years, AZD1222, BBIBP-CorV, and mRNA-1237 were the most effective candidate vaccines. *Conclusion*: All the candidate vaccines induced significant levels of SARS-CoV-2 neutralizing antibodies, but only AZD1222 and mRNA-1237 were certainly tested in patients aged ≥70 years. Compared with AZD1222, BNT162b and mRNA-1237 have the advantage that they can be quickly re-engineered to mimic new mutations of SARS-CoV-2.

## 1. Introduction

Vaccination represents one of the greatest medical advances of modern civilization [1]. As the coronavirus disease 2019 (COVID-19) pandemic continues to rage, the development of an effective vaccine is central to prevent further disease morbidity and mortality and, hopefully, limit the global spread of viral infection [2]. Research and testing of promising severe acute respiratory syndrome coronavirus 2 (SARS-CoV-2) vaccines has progressed at an unprecedented pace, with 63 candidates currently at clinical stage [3] by use of a broad range of technology platforms, from traditional to new-generation approaches [4].

As of 7 January 2021 SARS-CoV-2 vaccines appear to be in Phase III development according to the World Health Organization (WHO) [3], but in actual fact, 17 of them are being evaluated in Phase III trials, as four candidates are currently being tested in Phase II segment of a combined Phase II/III trial [5,6,7,8,9]. Six SARS-CoV-2 vaccines have been granted emergency use and/or full marketing authorization by the regulatory authorities, namely AZD1222, BBIBP-CorV, BBV-152, BNT162b2, mRNA-1237, and Sputnik V.

On 30 December, the United Kingdom and Argentina issued emergency use approval for AZD1222 [10,11], and on 3 January, India and Mexico followed suit [12,13]. China, the United Arab Emirates, and Bahrain fully approved BBIBP-CorV, while Egypt gave emergency use validation [14,15,16]. BBV-152 was approved for emergency use by the Indian government on 3 January [17]. BNT162b2 was granted emergency use authorization in the United States [18], Argentina [19], Chile, Ecuador [20], Costa Rica [21], Kuwait [22], Mexico [23], Panama, Singapore [24], and the European Union [25], whereas full approval was given to it in Bahrain, Canada, Saudi Arabia, and Switzerland [26,27,28,29]. mRNA-1237 is the second SARS-CoV-2 vaccine authorized for emergency use by the Food and Drug Administration, and Canada granted it full approval [30,31], while in January, Israel and the European Union gave emergency use authorization [32,33]. Sputnik V received emergency regulatory approval by Russia, Belarus, and Argentina [34,35]. 

Assessment of vaccine efficacy is particularly challenging when it comes to SARS-CoV-2, as fundamental understanding of the pathogen is constantly progressing [36]. Furthermore, despite the current approvals, there is still a paucity of published data concerning Phase III trials on candidate SARS-CoV-2 vaccines [37,38,39]. 

Therefore, since to date, there are no studies providing a head-to-head comparison across SARS-CoV-2 vaccines, the aim of this network meta-analysis was to compare the efficacy of candidate vaccines currently in Phase III clinical development in inducing neutralizing antibodies against SARS-CoV-2.

## 2. Materials and Methods

### 2.1. Protocol and Registration

**Figure 1:**
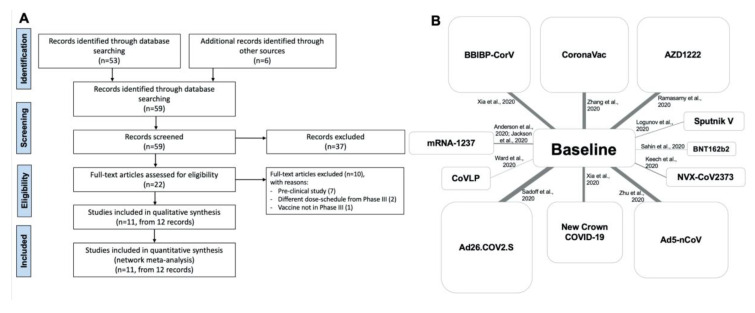
PRISMA-P flow diagram (**A**) and network nodes displaying the geometry of the network across candidate SARS-CoV-2 vaccines (**B**). The links between the nodes indicate the direct comparison across SARS-CoV-2 vaccines vs. baseline. The thickness of the lines is proportional to the number of vaccine recipients, and the area of the boxes is proportional to the number of subjects receiving the same SARS-CoV-2 vaccine. PRISMA-P: Preferred Reporting Items for Systematic Review and Meta-Analysis Protocols; SARS-CoV-2: severe acute respiratory syndrome coronavirus 2.

This quantitative synthesis was registered in the international prospective register of systematic reviews (PROSPERO, registration ID: CRD42020227062, available from https://www.crd.york.ac.uk/prospero/display_record.php?ID=CRD42020227062) and performed in agreement with the Preferred Reporting Items for Systematic Reviews and Meta-Analyses Protocols (PRISMA-P) [40]. The relative flow diagram and network nodes are shown in Figure 1A,B. This study satisfied all the recommended items reported by the PRISMA for network meta-analysis (PRISMA-NMA) checklists (Table S1) [41]. 

### 2.2. Eligibility Criteria

A comprehensive literature search was performed for clinical trials (both randomized and non-randomized) written in English and evaluating the level of neutralizing antibodies against SARS-CoV-2 of candidate vaccines that have reached Phase III clinical development. As an example, Table S2 reports the literature search terms used for OVID MEDLINE.

### 2.3. Information Sources

The search was performed in ClinicalTrials.gov (Bethesda, MD, USA), the Cochrane Central Register of Controlled Trials (CENTRAL), Embase, the EU Clinical Trials Register, MEDLINE, Scopus, and Web of Science to provide relevant studies published up to 18 December 2020. 

### 2.4. Search

The research string was as follows: (BBIBP-CorV OR BNT162b2 OR ((New Crown COVID-19) OR (SARS-COV-2 inactivated vaccine)) OR ((Sputnik V) OR Gam-COVID-Vac) OR (CoronaVac OR (adsorbed COVID-19 inactivated vaccine)) OR NVX-CoV2373 OR (AZD1222 OR (ChAdOx1 nCoV-19)) OR Ad5-nCoV OR (Ad26.COV2.S OR JNJ-78436735 OR Ad26COVS1 OR VAC31518) OR CoVLP OR mRNA-1273 OR INO-4800 OR COVISHIELD OR (RBD-dimer vaccine)) AND (antibody). Other sources selected to provide for relevant studies included the “Draft landscape of COVID-19 candidate vaccines,” released by WHO [42], and the online archive and distribution server of preprints MedxRiv (available at https://www.medrxiv.org) (accessed on 18 December 2020). Citations of previously published reviews were checked to identify further pertinent clinical trials, if any [43].

Literature search results were uploaded to Eppi-Reviewer 4 (EPPI-Centre Software, London, UK), a web-based software program for managing and analyzing data in literature reviews that facilitates collaboration among reviewers during the study selection process. 

### 2.5. Study Selection

Clinical trials that enrolled healthy adult volunteers and included at least one arm assessing the neutralizing antibody response of SARS-CoV-2 vaccines were included in the network meta-analysis. The studies in which the immunization schedule, dosing, and route of administration were consistent with those of Phase III studies, completed or ongoing, were selected and included in the network meta-analysis. 

Three reviewers independently examined the studies, and any difference in opinion concerning the selection of relevant clinical trials from literature searches and databases was resolved by consensus. 

### 2.6. Data Collection Process

Data from the clinical trials included in this quantitative synthesis were extracted from published papers, either peer-reviewed or preprint, and/or supplementary files. The data were checked and extracted for study characteristics and duration, pharmaceutical company, type of candidate SARS-CoV-2 vaccine with immunization schedule, dosing and route of administration, number of vaccine recipients, age, gender, and items to assess the Cochrane Risk of Bias 2 (RoB 2) [44]. 

The data were extracted in agreement with Data Extraction for Complex Meta-Analysis (DECiMAL) recommendations [45], and the level of SARS-CoV-2 neutralizing antibodies at different time points, including those at peak effect. When needed, the arithmetic mean and standard deviation were calculated from the geometric mean, median, range, and sample size, as previously described [46]. The inter- and intra-rater reliability for data abstraction was assessed via the Cohen’s kappa score, as previously described [47]. Briefly, Cohen’s kappa ≥0.80 indicated excellent agreement, coefficients between 0.61 and 0.80 substantial agreement, coefficients between 0.41 and 0.61 moderate agreement, and coefficients <0.41 fair to poor agreement. 

### 2.7. Data Items

The patient problem, intervention, comparison, and outcome (PICO) framework was applied to develop the literature search strategy, as previously reported [48]. The “Patient problem” was the humoral protection against SARS-CoV-2; the “Intervention” was the SARS-CoV-2 vaccines; the “Comparison” was performed across candidate SARS-CoV-2 vaccines and with respect to baseline; and the assessed “Outcome” was the level of neutralizing antibodies.

### 2.8. Endpoints

The primary endpoint of this meta-analysis was to compare the peak neutralizing antibody levels across candidate SARS-CoV-2 vaccines and compared to baseline. The secondary endpoint was to assess the time course of the neutralizing antibody response induced by the candidate SARS-CoV-2 vaccines after the last inoculation.

### 2.9. Summary Measures

Since the investigated studies assessed the same outcome (the level of SARS-CoV-2 neutralizing antibodies) by using different metrics and methods, the results of the network meta-analysis expressed as relative effect (RE) and 95% credible interval (95% CrI) were converted into the standardized mean difference (SMD = (difference in mean outcome between groups) × (standard deviation of outcome among participants) −1). The SMD was also reported in agreement with the rules of thumb proposed by Cohen and the Cochrane Collaboration [49,50]—namely, ≤0.5 represents a small effect, >0.5 to ≤0.8 a moderate effect, >0.8 to ≤1.3 a large effect, and >1.3 a very large effect. 

The probability that each intervention arm was the most effective/safe was calculated by counting the proportion of iterations of the chain in which each intervention arm had the best relative effect, and the surface under the cumulative ranking curve analysis (SUCRA), representing the summary of these probabilities [51]. The SUCRA is 1 when a treatment is considered to be the best, and 0 when a treatment is considered to be the worst [51]. The area under the curve (AUC), time to peak, and onset of action (*t*_1/2_) analyses were carried out for the time course levels of neutralizing antibody response induced by the candidate SARS-CoV-2 vaccines investigated for at least 4 weeks after the last inoculation.

### 2.10. Data Synthesis and Analysis

A network meta-analysis was performed to indirectly compare the peak levels of neutralizing antibodies across candidate SARS-CoV-2 vaccines. Subset analyses were performed in agreement with the average vaccine recipients’ age at baseline and according to the type of candidate vaccines. A full Bayesian evidence network was used in the network meta-analysis (chains: 4; initial value scaling: 2.5; tuning iterations: 20,000; simulation iterations: 50,000; tuning interval: 10). The convergence diagnostics for consistency and inconsistency were assessed via the Brooks–Gelman–Rubin method, as previously described [52]. Due to the characteristics of parameters besides the available data, the just proper non-informative distributions specified the prior densities, in agreement with the Bayesian Approaches to Clinical Trials and Health-Care Evaluation [53,54]. Since the distributions were sufficiently vague, the reference treatment, study baseline effects, and heterogeneity variance were unlikely to have a noticeable impact on model results. In this condition, the GeMTC software (developed by Gert van Valkenhoef et al., Groningen, Netherlands) automatically generates and runs the required Bayesian hierarchical model and selects the prior distributions and starting values as well, by heuristically determining a value for the outcome scale parameter (i.e., outcome scale S) [55,56]. The posterior mean deviance of data points in the unrelated mean effects model was plotted against their posterior mean deviance in the consistency model to provide information for identifying the loops in the treatment network where evidence was inconsistent [57]. 

### 2.11. Quality of Studies, Risk Bias, and Evidence Profile

The summary of the risk of bias for each included clinical trial was analyzed via the RoB 2 [44]. The weighted assessment of the risk of bias was analyzed via the Cochrane RoB 2 [44]. The risk of bias was performed for the primary endpoint, and it was checked via the normalized consistency/inconsistency analysis, a procedure that allows assessing whether the outcomes resulting from the consistency and inconsistency models fit adequately with the line of equality, as previously described [51]. The inconsistency of evidence was also investigated by quantifying the inconsistency factor that indicates whether one of the treatments had a different effect when it was compared with the others. Three reviewers independently assessed the quality of studies, risk bias, and evidence profile, and any difference in opinion was resolved by consensus.

The quality of the evidence was assessed for the primary endpoint in agreement with the Grading of Recommendations Assessment, Development, and Evaluation (GRADE) system, with ++++ indicating high quality of evidence, +++ moderate quality of evidence, ++ low quality of evidence, and + very low quality of evidence [58].

### 2.12. Software and Statistical Significance

The GeMTC software and OpenMetaAnalyst (CEBM, Brown University, Rhode Island, USA) were used to perform the meta-analysis [55,59], GraphPad Prism (San Diego, CA, USA) software to graph the data, GRADEpro GDT (McMaster University and Evidence Prime Inc., Hamilton, Canada) to assess the quality of evidence [58], and the robvis visualization software to perform the RoB 2 tool [60,61]. The statistical significance was set for *p* < 0.05. 

## 3. Results

### 3.1. Study Selection and Characteristics

The data obtained from 836 healthy adult recipients of candidate SARS-CoV-2 vaccines were extracted from 11 clinical studies (Table 1). The investigated candidate SARS-CoV-2 vaccines included four adenovirus-vector-based vaccines [62,63,64,65], three inactivated SARS-CoV-2 vaccines [66,67,68], two lipid nanoparticle-encapsulated mRNA-based vaccines [69,70,71], one SARS-CoV-2 recombinant spike glycoprotein nanoparticle vaccine [72], and one plant-derived virus-like particle vaccine [73]. The studies included in the network meta-analysis were two Phase I clinical trials [70,71,73], seven Phase I/II clinical trials [63,65,66,67,68,69,72], one Phase II randomized controlled trial (RCT) [64], and one Phase II/III RCT [62].

The inter-rater reliability for data abstraction was excellent before and after the learning process (Cohen’s kappa 0.96 and 1.00, respectively). The intra-rater reliability produced a Cohen’s kappa of 1.00 after the learning process.

### 3.2. Primary Endpoint

**Figure 2:**
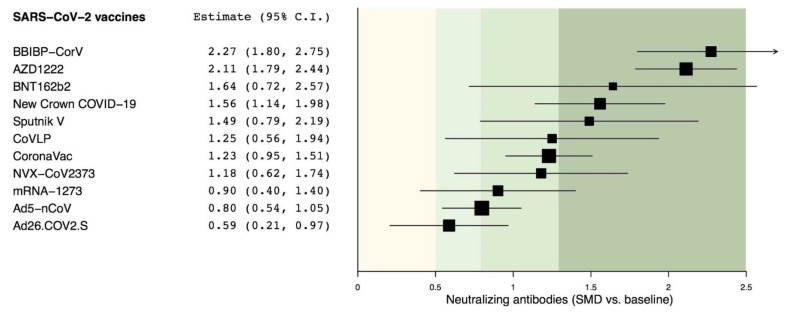
Overall forest plot of the impact of different candidate SARS-CoV-2 vaccines vs. baseline on the SMD in peak neutralizing antibodies. SARS-CoV-2 vaccine comparisons have been sorted in agreement with the level of efficacy; 95% CI: 95% confidence interval; SARS-CoV-2: severe acute respiratory syndrome coronavirus 2; SMD: standardized mean difference.

All candidate vaccines produced a significant (*p* < 0.05) peak level of neutralizing antibodies against SARS-CoV-2 with SMD effect estimates between 0.59 and 2.27 vs. baseline. The analysis of effect estimates indicated that BBIBP-CorV, AZD1222, BNT162b2, New Crown COVID-19, and Sputnik V induced a very large effect on the peak level of neutralizing antibodies against SARS-CoV-2 (SMD > 1.3); CoVLP, CoronaVac, NVX-CoV2373, and Ad5-nCoV induced a large effect (SMD > 0.8 to ≤1.3), whereas Ad26.COV2.S induced a medium effect (SMD > 0.5 to ≤0.8). Detailed SMD and 95% CI values with graphical data are shown as a forest plot in Figure 2. 

**Figure 3:**
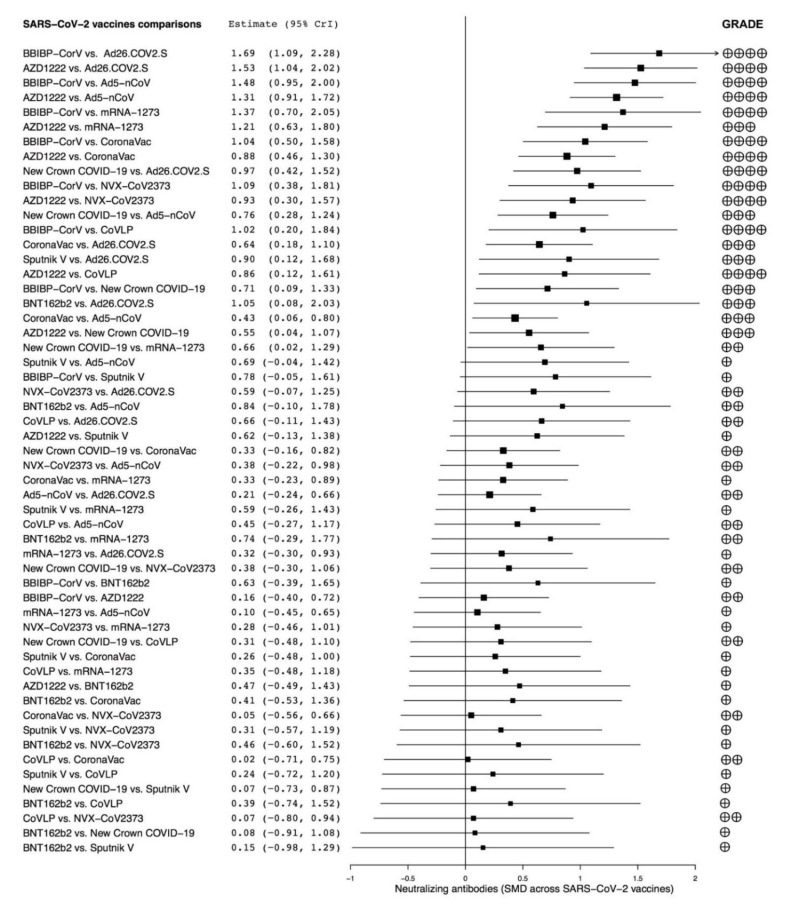
Overall forest plot of the comparisons across different candidate SARS-CoV-2 vaccines on the SMD in peak neutralizing antibodies and quality of evidence assessed via GRADE. SARS-CoV-2 vaccine comparisons have been sorted in agreement with the level of efficacy; 95% CrI: 95% credible interval; GRADE: Grading of Recommendations Assessment, Development, and Evaluation; SARS-CoV-2: severe acute respiratory syndrome coronavirus 2; SMD: standardized mean difference.

The network meta-analysis reported that BBIBP-CorV and AZD122 were significantly (*p* < 0.05) more effective at producing peak neutralizing antibodies than Ad26.COV2.S, Ad5–nCoV, mRNA-1237, CoronaVac, NVX–CoV2373, CoVLP, and New Crown COVID-19. New Crown COVID-19 was significantly (*p* < 0.05) more effective than Ad26.COV2.S, Ad5–nCoV, and mRNA-1237, whereas CoronaVac was significantly (*p* < 0.05) more effective than Ad26.COV2.S and Ad5–nCoV. Sputnik V and BNT162b2 were both significantly (*p* < 0.05) more effective than Ad26.COV2.S. The forest plot of the comparisons across the investigated SARS-CoV-2 vaccines is shown in Figure 3. 

**Figure 4:**
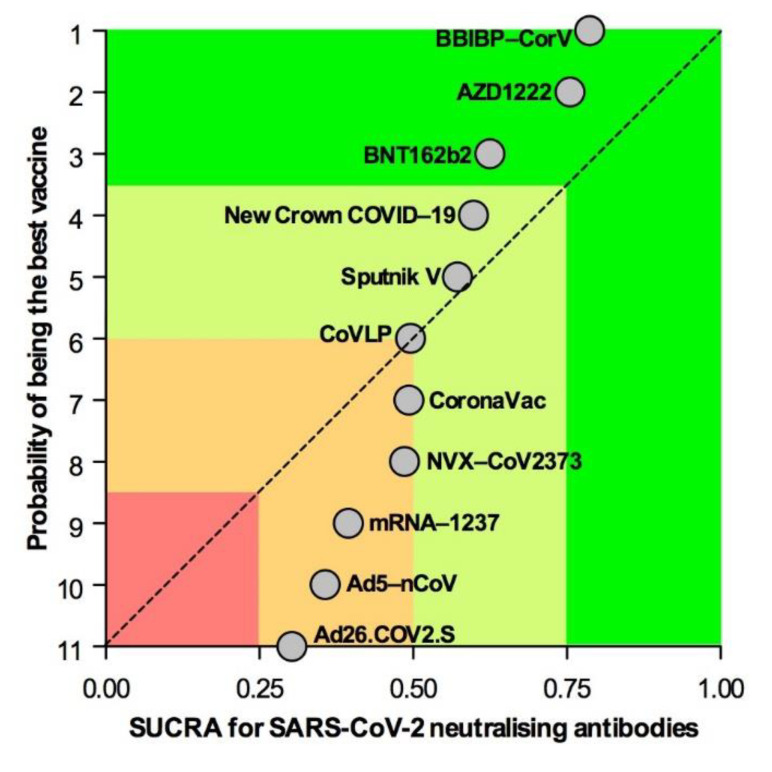
Overall ranking plot displaying the efficacy of candidate SARS-CoV-2 vaccines at inducing peak neutralizing antibody response. Vaccination strategies were plotted on the *x* axis according to SUCRA, where 1 results for a vaccine considered to be the best, and 0 for a vaccine considered to be the worst. SARS-CoV-2 vaccines were plotted on the *y* axis according to the rank probability of the best vaccine, where a score of 1 is assigned to the best vaccination strategy. SARS-CoV-2: severe acute respiratory syndrome coronavirus 2; SUCRA: surface under the cumulative ranking curve analysis.

The SUCRA showed that BBIBP-CorV, AZD1222, and BNT162b2 were the most effective candidate vaccines at producing peak SARS-CoV-2 neutralizing antibodies (1st quartile), followed by New Crown COVID-19 and Sputnik V (2nd quartile), CoVLP, CoronaVac, and NVX-CoV-2373 (borderline 2nd/3rd quartile), and mRNA, Ad5–nCoV, and Ad26.COV2.S (3rd quartile) (Figure 4). 

### 3.3. Subset Analyses

Subset analyses were performed in recipients of candidate SARS-CoV-2 vaccines aged ≤60 years and ≤70 years. The SUCRA indicated that in vaccine recipients ≤60 years old, AZD1222, BBIBP-CorV, and mRNA-1237 were the most effective candidate vaccines at producing peak SARS-CoV-2 neutralizing antibodies (1st quartile), followed by Ad26.COV2.S, BNT162b2, and New Crown COVID-19 (2nd quartile), Sputnik V (borderline 2nd/3rd quartile), and CoVLP, CoronaVac, NVX-CoV-2373, and Ad5–nCoV (3rd quartile) (Figure S1A). The SUCRA performed for vaccine recipients ≤70 years old confirmed the results obtained in those aged ≤60 years (Figure S1B). 

A further SUCRA performed according with the type of candidate vaccines indicated that in recipients aged either ≤60 years or ≤70 years, adenovirus-vector-based, mRNA-based, and inactivated SARS-CoV-2 vaccines were the best treatments at inducing peak neutralizing antibody response, followed by the less effective plant-derived virus-like particle and SARS-CoV-2 recombinant spike glycoprotein nanoparticle vaccines (Table S3).

### 3.4. Secondary Endpoint

The time course of the neutralizing antibody response to candidate SARS-CoV-2 vaccines is reported in Figure S2. Only BNT162b2 was investigated for nine weeks post last inoculation; Ad26.COV2.S, Ad5–nCoV, AZD1222, BBIBP-CorV, and CoronaVac were studied for four weeks; whereas the clinical trials on CoVLP, mRNA-1237, New Crown COVID-19, NVX-CoV-2373, and Sputnik V lasted less than three weeks.

To provide consistent and homogeneous findings, the analysis of the secondary endpoint was limited to vaccine recipients ≤60 years old, thus preventing spurious results due to age-related discrepancies across the included studies. Thus, Table S4 reported the AUC, time to peak, and *t*_1/2_ analyses of neutralizing antibody response to candidate SARS-CoV-2 vaccines investigated for four weeks post last inoculation. 

### 3.5. Risk of Bias and Quality of Evidence

The weighted plot for the assessment of the overall risk of bias by domains is shown in Figure S3, and the traffic light plot for the assessment of each included study is reported in Figure S4. Ten studies out of 11 had a low risk of bias for deviations from intended intervention (10 [90.9%]), missing outcome data (10 [90.9%]), and measurement of the outcomes (10 [90.9%]). Three studies had a high risk of bias for the randomization process (3 [27.3%]) and selection of the reported results (3 [27.3%]), whereas one study had some concerns in the domain of deviations from intended intervention (1 [9.1%]) and measurement of the outcomes (1 [9.1%]). 

The normalized consistency/inconsistency analysis indicated that all points fit adequately with the line of equality (*R*^2^ 0.995, slope 0.968, 95%CI 0.956–0.980) (Figure S5). The lack of bias in the overall Bayesian network was confirmed by the absence of significant (*p* > 0.05) inconsistency factors when the investigated candidate SARS-CoV-2 vaccines were compared indirectly. 

The assessment of the quality of evidence carried out via the GRADE system for the comparison across the candidate SARS-CoV-2 vaccines is shown in Figure 3. Overall, the quality of evidence was not affected by non-peer-reviewed publications. 

## 4. Discussion

The overall results of this network meta-analysis performed with no age restriction of recipients provide the high-quality evidence that four SARS-CoV-2 vaccines, namely AZD1222, BBIBP-CorV, BNT162b2, and New Crown COVID-19, elicite a very large effect in inducing the synthesis of neutralizing antibodies, and that AZD1222 and BBIBP-CorV are generally more effective than most of the other vaccines on this outcome. 

While the overall SUCRA ranked AZD1222, BBIBP-CorV, and BNT162b2 in the 1st efficacy quartile, the subset analysis in recipients of ≤60 and ≤70 years of age confirmed this finding only for AZD1222 and BBIBP-CorV. This evidence resulted also by the time-point analysis of vaccines investigated for at least four weeks, reporting that AZD1222 and BBIBP-CorV are those with the greatest AUC, with AZD1222 resulting in the fastest onset of action of only six days post last inoculation. In any case, the level of neutralizing antibodies was investigated for more than four weeks only for BNT162b2, resulting in stability at nine weeks post last inoculation. Moreover, although mRNA-1273 was ranked in the 1st quartile of SUCRA in recipients aged ≤60 and ≤70 years, unfortunately it was tested for only two weeks.

Interestingly, considering the type of vaccine, this network meta-analysis suggests that adenovirus-vector-based, mRNA-based, and inactivated SARS-CoV-2 vaccines are superior to the plant-derived virus-like particle and SARS-CoV-2 recombinant spike glycoprotein nanoparticle vaccines in inducing the synthesis of neutralizing antibodies, at least in subjects aged ≤60 and ≤70 years. 

In this respect, the question of age seems to be relevant in this quantitative synthesis. In fact, across the investigated vaccines, only the studies on AZD1222 and mRNA-1273 clearly reported to have quantified the level of neutralizing antibodies in recipients older than 70 years [62,70,71], whereas the number of patients older than 70 years was not clearly reported in the studies on Ad5-nCoV and Ad26.COV2.S [64,65]. Conversely, all the other studies on BBIBP-CorV, BNT162b2, New Crown COVID-19, Sputnik V, CoVLP, CoronaVac, and NVX-CoV2373 were performed in subjects aged ≤60 years. Therefore, in the light of these considerations on age and the rank resulting from the SUCRA, it can be assumed that AZD1222 should represent the first choice to elicit the production of neutralizing antibodies in subjects of any age, whereas BBIBP-CorV and mRNA-1237 have been proven to be as effective as AZD1222 only in recipients aged ≤60 years. Therefore, we cannot exclude that there may be an age-related specificity in the protective humoral response of vaccines against SARS-CoV-2.

In addition to the age of the subjects included in the studies, the number of enrolled recipients may also have modulated the effect estimates. In this respect, we cannot omit that when compared with the studies on BBIBP-CorV, AZD1222, New Crown COVID-19, CoronaVac, Ad5-nCoV, and Ad26.COV2.S [62,64,65,66,67,68], those on BNT162b2, Sputnik V, CoVLP, NVX-CoV2373, and mRNA-1273 were performed on few subjects [63,69,70,71,72,73], leading to potential bias due to the small-study effect, in which the treatment effect may be greater than that resulting in larger studies [74]. However, although some concerns on the quality of the studies resulted also from the Cochrane RoB 2 tool, the analysis of consistency and inconsistency suggested the lack of significant bias in the overall Bayesian network.

Certainly, the indirect comparison across different treatments of variables assessed by using different metrics and methods to measure the same outcome may be challenging. For this reason, in this network meta-analysis the change from baseline in the level of neutralizing antibodies was not reported as a simple summary estimate, such as the mean difference [75]. Conversely, the effect estimates were converted to SMD, a statistical tool that permits to combine and compare data that are presented in different units [75]. This statistical approach, already used in previous meta-analyses on candidate vaccines and supported by Cochrane [49,50,76], can also permit to assess the extent of the effect estimate.

It has been recognized that while the mean difference provides information in clinical units, SMD gives information in statistical units [75]. Indeed, this is the main limitation of our network meta-analysis. However, we have to highlight that vaccine-elicited neutralizing antibodies specific for SARS-CoV-2 play a pivotal role in viral neutralization and clearance, and in several studies they strongly correlated with receptor-binding domain protein levels detected in COVID-19 patients [77]. Moreover, although the level of neutralizing antibodies does not represent the gold standard for assessing the clinical efficacy of a vaccine, certainly it can be considered a suitable biomarker to predict a favorable humoral immune response against SARS-CoV-2 infection [77]. Therefore, looking forward to future head-to-head RCTs across different vaccines against SARS-CoV-2, to date the only way to rank the efficacy of these medications is to indirectly compare the level of neutralizing antibodies via the SMD approach in a Bayesian evidence network.

The antigen-specific T cell response induced by the investigated vaccines may also contribute to their efficacy. However, such a T cell response may diverge from the levels and quality of SARS-CoV-2 neutralizing antibodies. In this regard, unfortunately, the only available data are those concerning a study on BNT162b1, a vaccine that is not currently in Phase III clinical development, and the results showed that most participants had T helper type 1 skewed T cell immune responses with receptor-binding domain-specific CD8+ and CD4+ T cell expansion [78]. However, the extent of response varied across individuals [78]. Probably, the antigen-specific T cell response should also be included in a metric of vaccine efficacy.

## 5. Conclusions

The evidence provided by this research should be correctly contextualized and interpreted with caution, especially with respect to the potential clinical implications. In this respect, although the efficacy of the adenovirus-vector-based vaccine AZD1222 in producing neutralizing antibodies regardless of the age of recipients is currently supported by evidence [62], other effective vaccines such as those based on mRNA (i.e., BNT162b2 and mRNA-1273) have the unquestionable advantage that they can be quickly re-engineered to mimic new mutations of SARS-CoV-2 and, thus, be ready for use in a few weeks [79]. Last but not the least, since head-to-head clinical trials on SARS-CoV-2 vaccines are not expected due to the emergency related with the COVID-19 pandemic, this network meta-analysis should be updated as soon as clinical results from Phase III RCTs on the efficacy profile of most of the vaccines that are currently administered in the worldwide population are available, namely AZD1222, BBIBP-CorV, BBV-152, BNT162b2, mRNA-1237, and Sputnik V.

## Figures and Tables

**Table 1:** Characteristics of the clinical studies included in the network meta-analysis.

Study and Year and Reference	Trial Number Identifier	Study Characteristics	Vaccine Developer	SARS-CoV-2 Vaccine (Dose and Route of Administration as in Phase III Development)	Type of Candidate Vaccine	Study Duration with Follow-Up (Weeks)	Number of Scheduled Doses (Timing of Inoculations) as in Phase III Development	Number of Vaccine Recipients	Characteristics of Vaccine Recipients	Age (Mean and Range)	Male (%)
**Xia et al., 2020 [66]**	ChiCTR2000032459	Phase I/II, single center, randomized, double-blind, placebo-controlled, parallel-group	Beijing Institute of Biological Products/Sinopharm	BBIBP-CorV (4 µg IM)	Inactivated SARS-CoV-2 vaccine	7	Prime and boost inoculation (0, 21 days)	112	Healthy adults with negative IgG and IgM to SARS-CoV-2, with no history of travelling to Hubei Province (China), regions outside of China, or regions with reported COVID-19 cases from December 2019, and no history of SARS-CoV-2 infection	41.7 (18.0–59.0)	47.0
**Ramasamy et al., 2020 [62]**	NCT04400838, ISRCTN15281137	Phase II/III, multicenter, randomized, single-blind, negative-controlled, parallel-group	University of Oxford/AstraZeneca	AZD1222 or ChAdOx1 nCoV-19 or COVISHIELD (3.5–6.5 × 10^10^ viral particles IM)	Replication-defective chimpanzee adenovirus-vectored vaccine expressing full-length SARS-CoV-2 spike glycoprotein gene	8	Prime and boost inoculation (0, 28 days)	128	Healthy adults, seronegative to SARS-CoV-2 before enrolment, apart from those aged 18.0–55.0 years old	57.8 (19.0–83.0)	52.3
**Sahin et al., 2020 [69]**	NCT04380701, EudraCT: 2020-001038-36	Phase I/II, single center, non-randomized, open-label, dose-ranging, non-controlled, parallel-group	BioNTech/Fosun Pharma/Pfizer	BNT162b2 (30 µg IM)	3 LNP-encapsulated nucleoside-modified mRNA vaccine encoding trimerized SARS-CoV-2 RBD antigen of spike glycoprotein	12	Prime and boost inoculation (1, 22 days)	12	Healthy adults, with negative IgG and/or IgM to SARS-CoV-2, negative SARS-CoV-2 nucleic acid amplification test nasal swab, with no previous clinical or microbiological diagnosis of COVID-19, no receipt of medications to prevent COVID-19, and no previous vaccination with any coronavirus vaccine	46.7 (35.0–55.0)	66.7
**Xia et al., 2020 [67]**	ChiCTR2000031809	Phase I/II, single center, randomized, double-blind, placebo-controlled, parallel-group	Wuhan Institute of Biological Products/Sinopharm	New Crown COVID-19 (5 μg IM)	Inactivated SARS-CoV-2 vaccine	5	Prime and boost inoculation (0, 21 days)	84	Healthy adults with no history of SARS-CoV (via on-site inquiry) or SARS-CoV-2 infection (via serological test and PCR)	41.4 (18.0–59.0)	38.1
**Logunov et al., 2020 [63]**	NCT04436471	Phase I/II, single center, non-randomized, open-label, non-controlled, parallel-group	Gamaleya Research Institute	Sputnik V or Gam-COVID-Vac (1 × 10^11^ viral particles IM)	Recombinant adenovirus type 26 vector plus recombinant adenovirus type 5 vector carrying the gene for SARS-CoV-2 full-length spike glycoprotein	6	Prime and boost inoculation (0, 21 days)	20	Healthy adults with negative PCR and IgG and IgM to SARS-CoV-2, with no history of COVID-19 or contact with COVID-19 patients	26.4 (18.0–60.0)	70.0
**Ward et al., 2020 [73]**	NCT04450004	Phase I, single center, randomized, partially-blinded, dose-escalation, non-controlled, parallel-group	Medicago Inc./GlaxoSmithKline	CoVLP (3.75 μg IM)	Plant-derived virus-like particle vaccine adjuvanted with AS03	6	Prime and boost inoculation (0, 21 days)	20	Healthy adults with negative IgG and IgM to SARS-CoV-2, with not known current or previous laboratory-confirmed SARS-CoV-1 or SARS-CoV-2/COVID-19	34.7 (19.0–49.0)	25.0
**Zhang et al., 2020 [68]**	NCT04352608	Phase I/II, single center, randomized, double-blind, placebo-controlled, parallel-group	Sinovac	CoronaVac (3 μg IM)	Inactivated SARS-CoV-2 vaccine	6	Prime and boost inoculation (0, 14 days)	120	Healthy adults with negative PCR and negative IgG and IgM to SARS-CoV-2, with no history of contact with patients with SARS-CoV-2, or travelling or residence in Wuhan city and surrounding areas or other communities with case reports within 2 weeks before enrolment	42.0 (18.0–59.0)	45.0
**Keech et al., 2020 [72]**	NCT04368988	Phase I/II, single center, randomized, observer-blind, placebo-controlled, parallel-group	Novavax	NVX-CoV2373 (5 μg IM)	Full-length recombinant SARS-CoV-2 glycoprotein nanoparticle vaccine adjuvanted with Matrix M1	5	Prime and boost inoculation (0, 21 days)	26	Healthy adults with no history of SARS or COVID-19 or with negative PCR or ELISA, with no history of contact with SARS-COV-2 subjects or working in an occupation at high risk for exposure to SARS-CoV-2	29.5 (18.0–59.0)	50.0
**Anderson et al., 2020 [71]; Jackson et al., 2020 [71]**	NCT04283461	Phase I, single center, non-randomized, open-label, dose-escalation, parallel-group	Moderna/National Institute of Allergy and Infectious Diseases’ Vaccine Research Center	mRNA-1273 (100 μg IM)	LNP-encapsulated nucleoside-modified mRNA vaccine encoding SARS-CoV-2 prefusion-stabilized full-length spike glycoprotein trimer	6	Prime and boost inoculation (0, 28 days)	35	Healthy adults, not screened for current or past SARS-CoV-2 infection by PCR or serology before enrolment	55.9 (18.0–55.0; 56.0–70.0: ≥71.0)	42.3
**Zhu et al., 2020 [64]**	NCT04341389	Phase II, single center, randomized, double-blind, placebo-controlled, parallel-group	CanSino Biological Inc./Beijing Institute of Biotechnology	Ad5-nCoV (5 × 10^10^ viral particles IM)	Recombinant adenovirus type 5 vector vaccine	4	Single inoculation	129	Healthy adults, HIV-negative, with no history of SARS-CoV-2 infection, confirmed by negative SARS-CoV-2 fingertip rapid blood test	39.7 (18.0–44.0; 45.0 –54.0; ≥55.0)	50.0
**Sadoff et al., 2020 [65]**	NCT04436276	Phase I/II, multicenter, randomized, double-blind (immunogenicity data were unblinded), placebo-controlled, parallel-group	Janssen Pharmaceutical Companies	Ad26.COV2.S or JNJ-78436735 or Ad26COVS1 (5 × 10^10^ viral particles IM)	Replication-defective recombinant adenovirus type 26 vector vaccine expressing SARS-CoV-2 stabilized prefusion spike glycoprotein	4	Single inoculation	150	Healthy adults with negative PCR to SARS-CoV-2	18.0–55.0; ≥65.0 (cohort 1a and 3)^a^	NA

a Immunogenicity data on cohort 56–64 years of age and cohort 1b not available. COVID-19: coronavirus disease 2019; ELISA: enzyme-linked immunosorbent assay; HIV: human immunodeficiency virus; IgG: immunoglobulin G; IgM: immunoglobulin M; IM: intramuscular; LNP: lipid nanoparticle; mRNA: messenger ribonucleic acid; NA: not available; PCR: polymerase chain reaction; RBD: receptor-binding domain; SARS-CoV-2: severe acute respiratory syndrome coronavirus 2.

## Data Availability

The data presented in this study are available in the article and Supplementary Materials.

## Supplementary Materials

The following are available online at https://www.mdpi.com/2076-393X/9/3/227/s1, Figure S1: Overall ranking plot of subset analyses displaying the efficacy of candidate SARS-CoV-2 vaccines at inducing peak neutralizing antibody response in vaccine recipients ≤60 years of age (A) and ≤70 years of age (B). Vaccination strategies were plotted on X-axis according to SUCRA, where 1 results for a vaccine considered to be the best, and 0 for a vaccine considered to be the worst. SARS-CoV-2 vaccines were plotted on Y-axis according to the rank probability of best vaccine, where a score of 1 is assigned to the best vaccination strategy. SARS-CoV-2: severe acute respiratory syndrome coronavirus 2; SUCRA: surface under the cumulative ranking curve analysis, Figure S2: Neutralizing antibody response to candidate SARS-CoV-2 vaccines monitored across different time-points post inoculation vs. baseline in healthy volunteers ≤60 years of age. SMD: standardized mean difference, Figure S3: Weighted plot for the assessment of the overall risk of bias via the Cochrane RoB 2 tool (n = 11 clinical trials). RoB 2: Risk of Bias 2, Figure S4:Traffic light plot for assessment of the risk of bias of each included clinical trial via the Cochrane RoB 2 tool. D1: bias arising from the randomization process; D2: bias due to deviations from intended intervention; D3: bias due to missing outcome data; D4: bias in measurement of the outcome; D5: bias in selection of the reported result. RoB 2: Risk of Bias 2, Figure S5: Publication bias assessment via the normalized consistency/inconsistency plot (linear regression and 95% prediction bands) of different SARS-CoV-2 vaccines with respect to the peak level of neutralizing antibodies. SARS-CoV-2: severe acute respiratory syndrome coronavirus 2, Table S1:PRISMA-P checklist, Table S2: Literature search terms used for OVID MEDLINE. The final search strategy applied to conduct this network meta-analysis is reported at step #29, Table S3; A subset analysis via SUCRA performed according with the age of recipients and the type of candidate SARS-CoV-2 vaccines, Table S4: AUC, time to peak, and t1/2 analyses of neutralizing antibody response to candidate SARS-CoV-2 vaccines investigated for 4 weeks post last inoculation in healthy recipients ≤60 years of age.
