# Supplementary material for: SARS-CoV-2 Neutralizing Antibodies: A Network Meta-Analysis across Vaccines
Source: Vaccines (Basel). 2021 Mar 5;9(3):227. doi: 10.3390/vaccines9030227 (PMC7999682; doi:10.3390/vaccines9030227)
Supplement: Supplementary file 1 [file vaccines-09-00227-s001.pdf]

SARS-CoV-2 neutralizing antibodies: a network meta-analysis across vaccines

Paola Rogliani MD[1], Alfredo Chetta MD[2], Mario Cazzola MD[1] and Luigino Calzetta PhD[2,*]

1. Unit of Respiratory Medicine, Department of Experimental Medicine, University of Rome “Tor Vergata”, Rome, Italy.
2. Department of Medicine and Surgery, Respiratory Disease and Lung Function Unit, University of Parma, Parma, Italy.

*Corresponding author: Luigino Calzetta, Department of Medicine and Surgery, Respiratory Disease and Lung Function Unit, University of Parma, Via Rasori 10, 43126 - Parma, Italy. E-mail: luigino.calzetta@unipr.it

Table S1. PRISMA-P checklist.

Section/topic	#	Checklist item	Reported on page #
**TITLE**			
Title	1	Identify the report as a systematic review, meta-analysis, or both.	1 main MS
**ABSTRACT**			
Structured summary	2	Provide a structured summary including, as applicable: background; objectives; data sources; study eligibility criteria, participants, and interventions; study appraisal and synthesis methods; results; limitations; conclusions and implications of key findings; systematic review registration number.	3-4 main MS
**INTRODUCTION**			
Rationale	3	Describe the rationale for the review in the context of what is already known.	5 main MS
Objectives	4	Provide an explicit statement of questions being addressed with reference to participants, interventions, comparisons, outcomes, and study design (PICOS).	6 main MS
**METHODS**			
Protocol and registration	5	Indicate if a review protocol exists, if and where it can be accessed (e.g., Web address), and, if available, provide registration information including registration number.	7 main MS
Eligibility criteria	6	Specify study characteristics (e.g., PICOS, length of follow-up) and report characteristics (e.g., years considered, language, publication status) used as criteria for eligibility, giving rationale.	7 main MS
Information sources	7	Describe all information sources (e.g., databases with dates of coverage, contact with study authors to identify additional studies) in the search and date last searched.	7-8 main MS
Search	8	Present full electronic search strategy for at least one database, including any limits used, such that it could be repeated.	8 main MS; Table S2
Study selection	9	State the process for selecting studies (i.e., screening, eligibility, included in systematic review, and, if applicable, included in the meta-analysis).	8 main MS
Data collection process	10	Describe method of data extraction from reports (e.g., piloted forms, independently, in duplicate) and any processes for obtaining and confirming data from investigators.	9 main MS
Data items	11	List and define all variables for which data were sought (e.g., PICOS, funding sources) and any assumptions and simplifications made.	9-10 main MS
Risk of bias in individual studies	12	Describe methods used for assessing risk of bias of individual studies (including specification of whether this was done at the study or outcome level), and how this information is to be used in any data synthesis.	11-12 main MS

Summary measures	13	State the principal summary measures (e.g., risk ratio, difference in means).	11 main MS
Synthesis of results	14	Describe the methods of handling data and combining results of studies, if done, including measures of consistency (e.g., I^2) for each meta-analysis.	11 main MS
Risk of bias across studies	15	Specify any assessment of risk of bias that may affect the cumulative evidence (e.g., publication bias, selective reporting within studies).	11-12 main MS
Additional analyses	16	Describe methods of additional analyses (e.g., sensitivity or subgroup analyses, meta-regression), if done, indicating which were pre-specified.	10 main MS
**RESULTS**			
Study selection	17	Give numbers of studies screened, assessed for eligibility, and included in the review, with reasons for exclusions at each stage, ideally with a flow diagram.	13 main MS; Figure 1
Study characteristics	18	For each study, present characteristics for which data were extracted (e.g., study size, PICOS, follow-up period) and provide the citations.	13 main MS; Table 1
Risk of bias within studies	19	Present data on risk of bias of each study and, if available, any outcome level assessment (see item 12).	15-16 main MS; Figure S4
Results of individual studies	20	For all outcomes considered (benefits or harms), present, for each study: (a) simple summary data for each intervention group (b) effect estimates and confidence intervals, ideally with a forest plot.	12 main MS; Figure 2
Synthesis of results	21	Present results of each meta-analysis done, including confidence intervals and measures of consistency.	14 main MS; Figure 3, 4
Risk of bias across studies	22	Present results of any assessment of risk of bias across studies (see Item 15).	15-16 main MS; Figure S3, S5
Additional analysis	23	Give results of additional analyses, if done (e.g., sensitivity or subgroup analyses, meta-regression [see Item 16]).	14-15 main MS; Table S3
**DISCUSSION**			

Summary of evidence	24	Summarize the main findings including the strength of evidence for each main outcome; consider their relevance to key groups (e.g., healthcare providers, users, and policy makers).	17 main MS
Limitations	25	Discuss limitations at study and outcome level (e.g., risk of bias), and at review-level (e.g., incomplete retrieval of identified research, reporting bias).	19 main MS
Conclusions	26	Provide a general interpretation of the results in the context of other evidence, and implications for future research.	20 main MS
**FUNDING**			
Funding	27	Describe sources of funding for the systematic review and other support (e.g., supply of data); role of funders for the systematic review.	22 main MS

MS: manuscript.

Table S2. Literature search terms used for OVID MEDLINE. The final search strategy applied to conduct this network meta-analysis is reported at step #29.

#	Search strategy
1	"BNT162b2".mp. [mp=ti, ab, tx, ct, sh, ot, nm, hw, fx, kf, ox, px, rx, an, ui, sy]
2	"BBIBP-CorV".mp. [mp=ti, ab, tx, ct, sh, ot, nm, hw, fx, kf, ox, px, rx, an, ui, sy]
3	"New Crown COVID-19".mp. [mp=ti, ab, tx, ct, sh, ot, nm, hw, fx, kf, ox, px, rx, an, ui, sy]
4	"SARS-COV-2 inactivated vaccine".mp. [mp=ti, ab, tx, ct, sh, ot, nm, hw, fx, kf, ox, px, rx, an, ui, sy]
5	"Sputnik V".mp. [mp=ti, ab, tx, ct, sh, ot, nm, hw, fx, kf, ox, px, rx, an, ui, sy]
6	"Gam-COVID-Vac".mp. [mp=ti, ab, tx, ct, sh, ot, nm, hw, fx, kf, ox, px, rx, an, ui, sy]
7	"CoronaVac".mp. [mp=ti, ab, tx, ct, sh, ot, nm, hw, fx, kf, ox, px, rx, an, ui, sy]
8	"adsorbed COVID-19 inactivated vaccine".mp. [mp=ti, ab, tx, ct, sh, ot, nm, hw, fx, kf, ox, px, rx, an, ui, sy]
9	"NVX-CoV2373".mp. [mp=ti, ab, tx, ct, sh, ot, nm, hw, fx, kf, ox, px, rx, an, ui, sy]
10	"AZD1222".mp. [mp=ti, ab, tx, ct, sh, ot, nm, hw, fx, kf, ox, px, rx, an, ui, sy]
11	"ChAdOx1 nCoV-19".mp. [mp=ti, ab, tx, ct, sh, ot, nm, hw, fx, kf, ox, px, rx, an, ui, sy]
12	"Ad5-nCoV".mp. [mp=ti, ab, tx, ct, sh, ot, nm, hw, fx, kf, ox, px, rx, an, ui, sy]
13	"Ad26.COV2.S".mp. [mp=ti, ab, tx, ct, sh, ot, nm, hw, fx, kf, ox, px, rx, an, ui, sy]
14	"JNJ-78436735".mp. [mp=ti, ab, tx, ct, sh, ot, nm, hw, fx, kf, ox, px, rx, an, ui, sy]
15	"Ad26COVS1".mp. [mp=ti, ab, tx, ct, sh, ot, nm, hw, fx, kf, ox, px, rx, an, ui, sy]
16	"VAC31518".mp. [mp=ti, ab, tx, ct, sh, ot, nm, hw, fx, kf, ox, px, rx, an, ui, sy]
17	"CoVLP".mp. [mp=ti, ab, tx, ct, sh, ot, nm, hw, fx, kf, ox, px, rx, an, ui, sy]
18	"mRNA-1273".mp. [mp=ti, ab, tx, ct, sh, ot, nm, hw, fx, kf, ox, px, rx, an, ui, sy]
19	"INO-4800".mp. [mp=ti, ab, tx, ct, sh, ot, nm, hw, fx, kf, ox, px, rx, an, ui, sy]
20	"COVISHIELD".mp. [mp=ti, ab, tx, ct, sh, ot, nm, hw, fx, kf, ox, px, rx, an, ui, sy]
21	"RBD-dimer vaccine".mp. [mp=ti, ab, tx, ct, sh, ot, nm, hw, fx, kf, ox, px, rx, an, ui, sy]
22	antibod*.mp. [mp=ti, ab, tx, ct, sh, ot, nm, hw, fx, kf, ox, px, rx, an, ui, sy]
23	3 or 4
24	5 or 6
25	7 or 8
26	10 or 11 or 20
27	13 or 14 or 15 or 16
28	1 or 2 or 9 or 12 or 17 or 18 or 19 or 21 or 23 or 24 or 25 or 26 or 27
29	22 and 28

Table S3. A subset analysis via SUCRA performed according with the age of recipients and the type of candidate SARS-CoV-2 vaccines.

	**Age of recipients**			
	**≤60**		**≤70**	
**Type of candidate vaccine**	**Quartile**	**SUCRA**	**Quartile**	**SUCRA**
mRNA-based	2nd	0·734	2nd	0·720
Adenovirus-vector-based	2nd	0·668	2nd	0·652
Inactivated SARS-CoV-2	2nd	0·652	2nd	0·664
Plant-derived virus-like particle	3rd	0·462	3rd	0·476
SARS-CoV-2 recombinant spike glycoprotein nanoparticle	3rd	0·440	3rd	0·448

SARS-CoV-2: severe acute respiratory syndrome coronavirus 2; SUCRA: surface under the cumulative ranking curve analysis.

Table S4. AUC, time to peak, and $t_{1/2}$ analyses of neutralizing antibody response to candidate SARS-CoV-2 vaccines investigated for 4 weeks post last inoculation in healthy recipients ≤60 years of age.

	**Ad26.COV2.S**	**Ad5-nCoV**	**AZD1222**	**BBIBP-CorV**	**BNT162b2**	**CoronaVac**
**AUC**	26·36±23·81	11·00±20·08	52·76±21·52	42·05±17·44	37·24±18·77	28·22±18·66
**Time to peak (days post last inoculation)**	29	28	14	28	7	28
**$t_{1/2}$ (days post last inoculation)**	14	14	6	8	4	7

Data are reported as mean±SEM. AUC: area under the curve; SARS-CoV-2: severe acute respiratory syndrome coronavirus 2; SEM: standard error of the mean; SMD: standardized mean difference; $t_{1/2}$: onset of action.

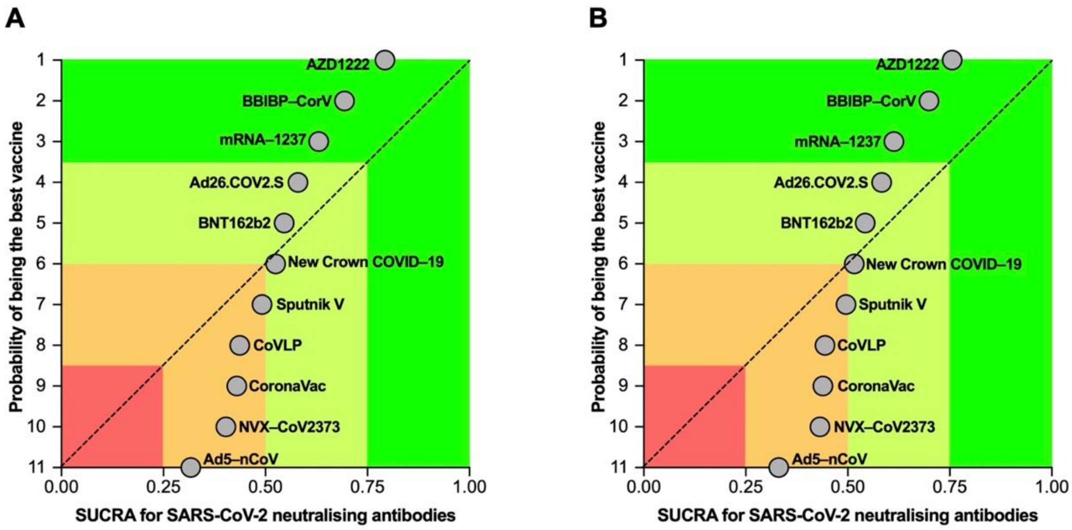

Figure S1. Overall ranking plot of subset analyses displaying the efficacy of candidate SARS-CoV-2 vaccines at inducing peak neutralizing antibody response in vaccine recipients ≤60 years of age (A) and ≤70 years of age (B). Vaccination strategies were plotted on X-axis according to SUCRA, where 1 results for a vaccine considered to be the best, and 0 for a vaccine considered to be the worst. SARS-CoV-2 vaccines were plotted on Y-axis according to the rank probability of best vaccine, where a score of 1 is assigned to the best vaccination strategy. SARS-CoV-2: severe acute respiratory syndrome coronavirus 2; SUCRA: surface under the cumulative ranking curve analysis.

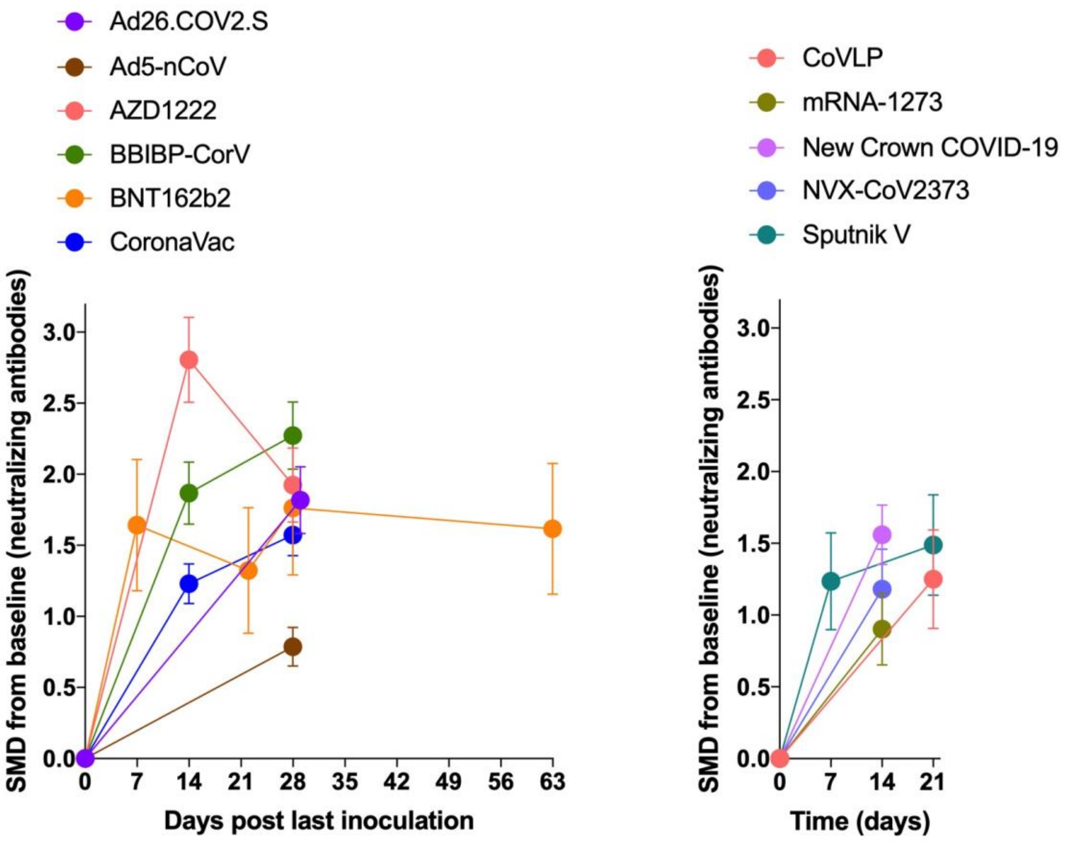

Figure S2. Neutralizing antibody response to candidate SARS-CoV-2 vaccines monitored across different time-points post inoculation vs. baseline in healthy volunteers ≤60 years of age. SMD: standardized mean difference.

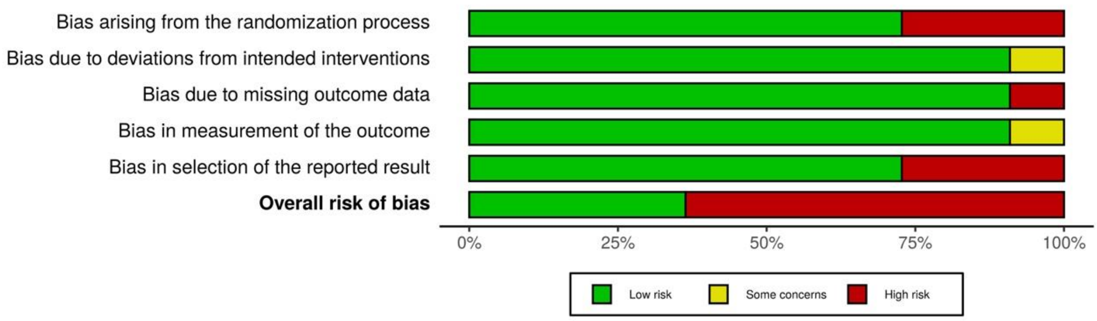

Figure S3. Weighted plot for the assessment of the overall risk of bias via the Cochrane RoB 2 tool (n=11 clinical trials). RoB 2: Risk of Bias 2.

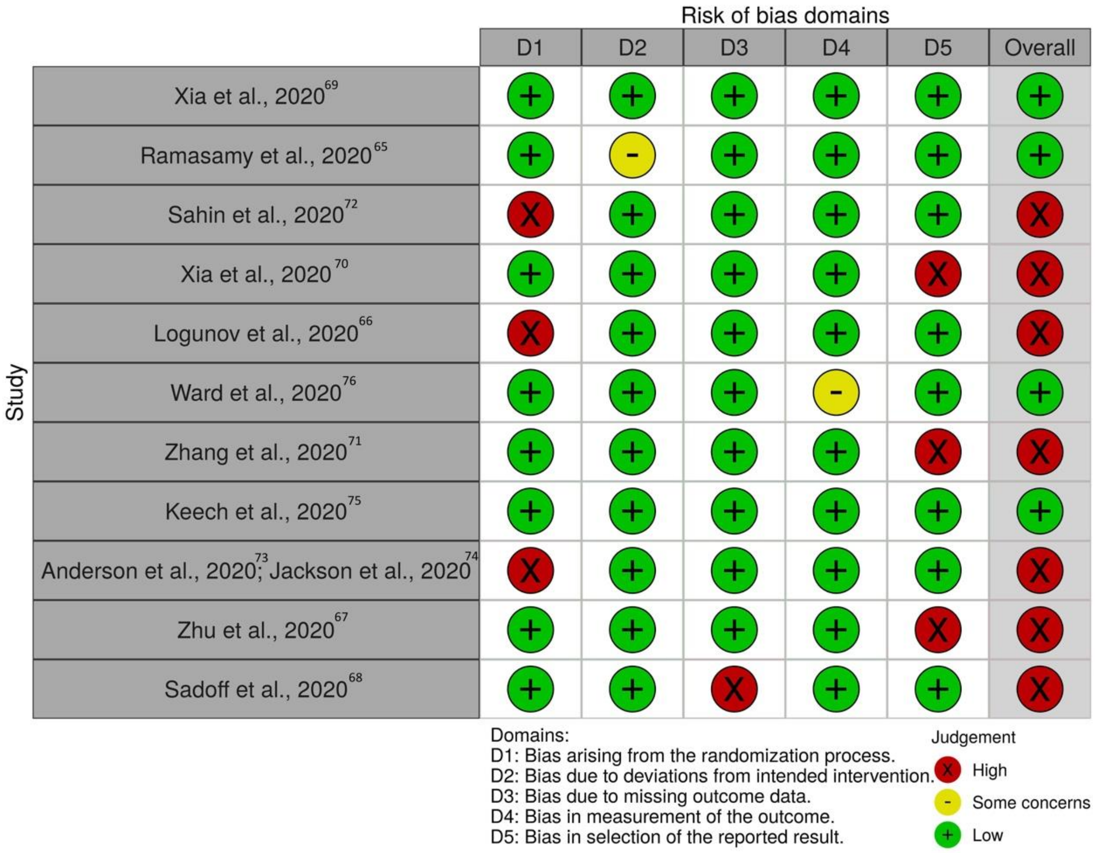

Figure S4. Traffic light plot for assessment of the risk of bias of each included clinical trial via the Cochrane RoB 2 tool. D1: bias arising from the randomization process; D2: bias due to deviations from intended intervention; D3: bias due to missing outcome data; D4: bias in measurement of the outcome; D5: bias in selection of the reported result. RoB 2: Risk of Bias 2.

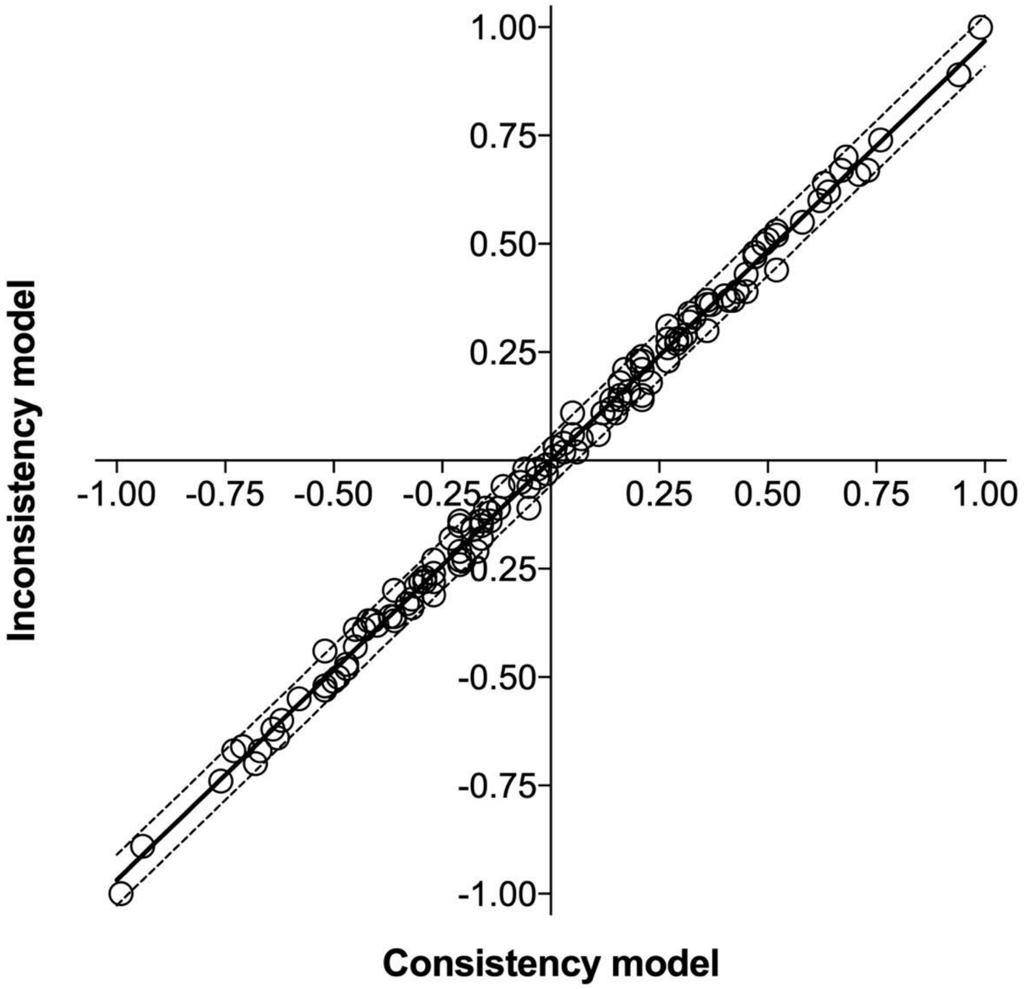

Figure S5. Publication bias assessment via the normalized consistency/inconsistency plot (linear regression and 95% prediction bands) of different SARS-CoV-2 vaccines with respect to the peak level of neutralizing antibodies. SARS-CoV-2: severe acute respiratory syndrome coronavirus 2.
